# Hemophagocytic Lymphohistiocytosis Triggered by Acute Interstitial Pneumonitis in a Young Woman With Rheumatoid Arthritis

**DOI:** 10.7759/cureus.85299

**Published:** 2025-06-03

**Authors:** Moemen Hasaballah, Mohamed Elsayed, Omar Shafey, Mohamed Abdulmajeed

**Affiliations:** 1 General Internal Medicine, Luton and Dunstable University Hospital, Luton, GBR; 2 General Internal Medicine, Addenbrooke's Hospital, Cambridge University Hospitals NHS Foundation Trust, Cambridge, GBR; 3 Gastroenterology, Royal Sussex County Hospital, Brighton, GBR

**Keywords:** acute interstitial pneumonitis, autoimmune disease trigger, ferritin elevation, hemophagocytic lymphohistiocytosis (hlh), rheumatoid arthritis, triglyceride elevation

## Abstract

Hemophagocytic lymphohistiocytosis (HLH) is an uncommon but potentially life-threatening condition where the body's immune system becomes overactive, causing widespread inflammation and organ damage. In this report, we present the case of a 21-year-old woman with a history of rheumatoid arthritis who developed secondary HLH, triggered by acute interstitial pneumonitis (AIP). This case sheds light on the importance of early recognition and intervention in managing this rare condition. It emphasizes the need to consider HLH in patients presenting with unexplained systemic inflammation similar to this case, particularly when triggered by AIP, as evidenced by the respiratory involvement in this case.

## Introduction

Hemophagocytic lymphohistiocytosis (HLH) is a rare disorder where the immune system goes into overdrive, leading to severe inflammation that can affect multiple organs. It can be genetic or triggered by infections, cancers, or autoimmune diseases. In some cases, it is linked to lung diseases, although this is less commonly reported [1,2].

Diagnosing HLH can be tricky. It often mimics infections or sepsis, and the symptoms may overlap with other serious conditions. Doctors usually follow the HLH-2004 criteria, which include signs like persistent fever, low blood counts, enlarged spleen, and very high ferritin levels [2]. Not every patient meets all the criteria, and waiting for every sign to appear can delay treatment [3].

We report a case where HLH appeared to be triggered by acute interstitial pneumonitis (AIP) in a young woman with a history of rheumatoid arthritis. Her story sheds light on the challenges in diagnosing HLH and the importance of acting quickly, even when all the typical markers are not present.

## Case presentation

A 21-year-old White British woman came to the hospital with a one-day history of feeling unwell. Her symptoms included continuous fever, headache, photophopia, diarrhea, dry cough, and abdominal pain. She had just returned from an 11-day trip to Sri Lanka. Her medical history included seropositive rheumatoid arthritis, joint hypermobility syndrome, and mental health conditions such as depression, anxiety, and PTSD. At the time of presentation, she was taking quetiapine and had previously used hydroxychloroquine for her arthritis, which was stopped nine months ago.

Socially, she smokes six cigarettes per day and drinks alcohol occasionally. She denied any recent sexual activity, drug use, tattoos, or consumption of raw food abroad. She also had no known family history of autoimmune or inflammatory conditions.

On examination, she was alert but appeared unwell. Her vital signs showed a temperature of 38°C, heart rate of 90 bpm, blood pressure of 99/61 mmHg, respiratory rate of 16, and oxygen saturation of 96% on room air. Chest auscultation revealed reduced air entry at the bases. Her abdomen was generally tender without guarding. Joint examination showed swelling in her left ankle, pain in her right ankle, and tenderness in both knees. There was no rash, clubbing, or signs of confusion.

Laboratory tests showed extremely high ferritin levels (7957 ng/mL), elevated triglycerides, anemia, and thrombocytopenia (Table 1).

**Table 1:** Laboratory test results.

Test	Result	Normal Range
Ferritin	7957 µg/L	13-150 µg/L
Triglycerides	3.4 mmol/L	0-1.6 mmol/L
Hemoglobin	89 g/L	120-160 g/L
Platelets	89×10⁹/L	150-450×10⁹/L
Fibrinogen	1.9 g/L	1.8-3.6 g/L
White blood cell count	8.4×10⁹/L	4-11×10⁹/L
ALT (alanine aminotransferase)	233 U/L	0-32 U/L
ALP (alkaline phosphatase)	172 U/L	30-130 U/L
AST (aspartate aminotransferase)	251 U/L	0-32 U/L
C-reactive protein	66 mg/L	0-4.9 mg/L
International normalized ratio (INR)	1.4	0.8-1.2
Albumin	18 g/L	35-50 g/L
Creatinine	109 µmol/L	44-80 µmol/L
LDH (lactate dehydrogenase)	669 U/L	0-249 U/L
C3 complement	0.41 g/L	0.9-1.8 g/L
C4 complement	0.07 g/L	0.1-0.4 g/L
Immunoglobulin G (IgG)	8.93 g/L	7-16 g/L
Immunoglobulin A (IgA)	2.50 g/L	0.7-4 g/L
Immunoglobulin M (IgM)	1.90 g/L	0.4-2.3 g/L
Anti-nuclear antibodies (HEp-2)	Negative	-
ANCA myeloperoxidase (MPO) antibodies	<0.3 kU/L	0-3.4 kU/L
ANCA serine proteinase 3 (PR3) antibodies	<0.7 kU/L	0-1.9 kU/L

The extensive infectious screening was negative, including tests for malaria, dengue, leptospirosis, syphilis, chikungunya, rickettsia, brucella, leishmania, parvovirus, flavivirus, strongyloides, toxoplasma, aspergillus, EBV, CMV, HIV, HSV, hepatitis viruses, tuberculosis (TB), legionella, mycoplasma, and COVID-19. Cerebrospinal fluid (CSF) analysis, including microscopy, culture, and biochemistry, was within normal limits.

**Figure 1:**
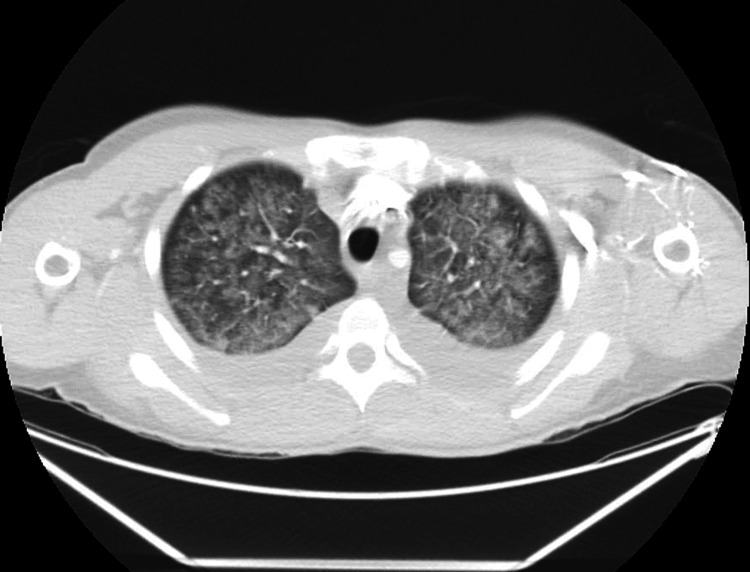
CT of the chest showing diffuse ground-glass opacities in the lungs.

**Figure 2:**
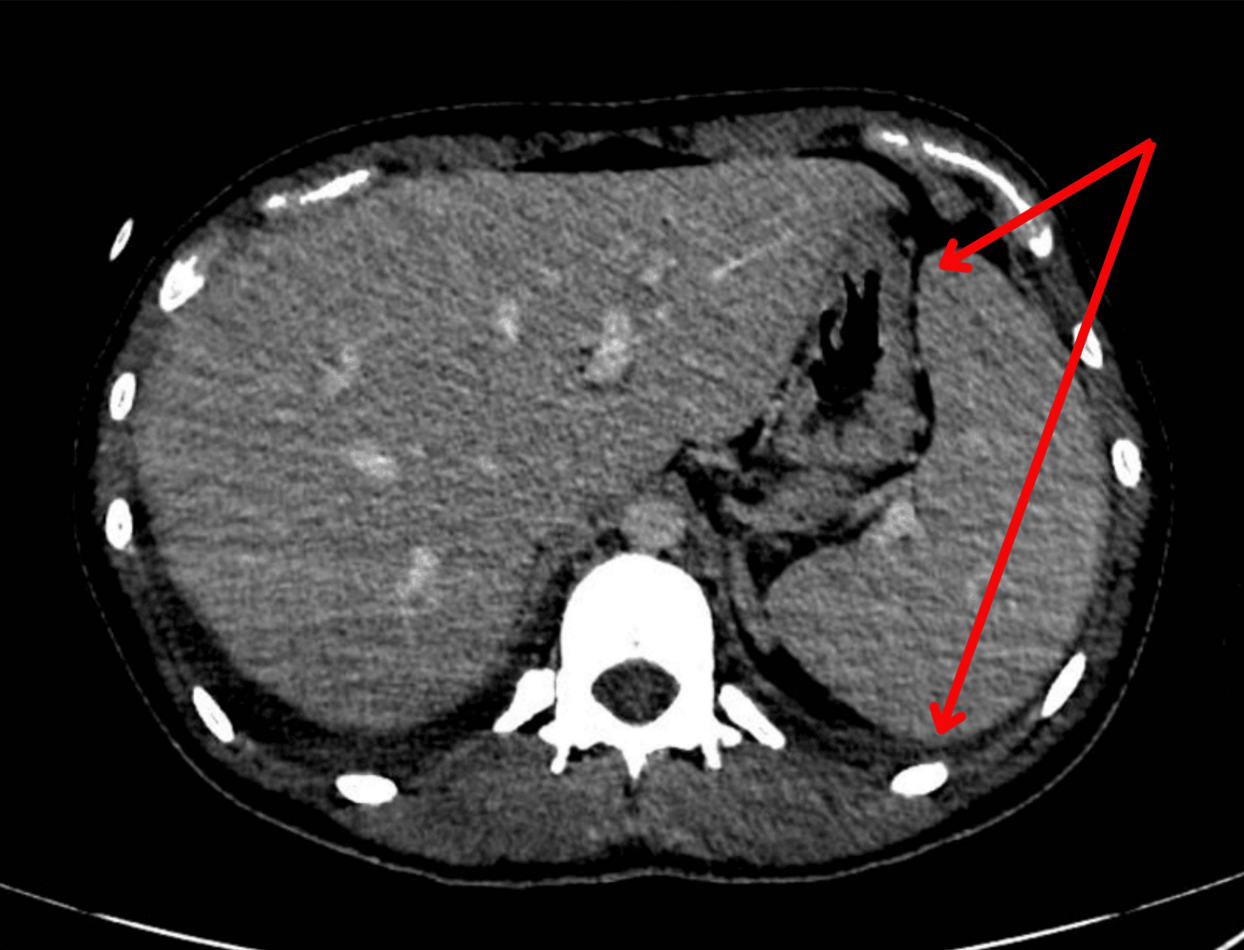
CT of the abdomen showing mild splenomegaly.

Further investigations, including a CT scan of the chest, revealed diffuse ground-glass opacities in the lungs, suggestive of AIP (Figure 1). A CT of the abdomen revealed mild splenomegaly (Figure 2). A bone marrow aspirate was performed later but showed no evidence of hemophagocytosis or hematological malignancy.

The patient was started on Meropenem on admission for possible sepsis. By the third day of hospitalization, her condition deteriorated significantly. She developed a high-grade fever approaching 40°C and required escalating oxygen support, eventually progressing to 15 L/min via a non-rebreather mask before deteriorating further to require 60% oxygen via a Venturi mask. Despite a broad initial workup, no infectious or autoimmune cause was found. Given her clinical picture, fever, cytopenias, high ferritin and triglycerides, and organ involvement, she was diagnosed with HLH, likely triggered by AIP.

Meropenem was discontinued, and treatment was initiated promptly with intravenous methylprednisolone at a dose of 500 mg daily for three days. Co-trimoxazole (160 mg/800 mg) was administered orally every other day for three weeks for prophylaxis. Anakinra was started at 200 mg twice daily for two days, followed by a tapering regimen over the subsequent weeks. Oral prednisolone was introduced after the intravenous course and gradually tapered thereafter. Remarkably, within a day of finishing high-dose steroids, her oxygen levels returned to normal and her symptoms began to resolve.

## Discussion

This case highlights an unusual cause of HLH in a young adult with no active infection or malignancy. While HLH is often linked to cancer, viral infections like Epstein-Barr virus, or autoimmune conditions such as lupus, this patient appeared to develop it in response to AIP [1,2].

HLH is a condition in which the immune system becomes excessively activated, leading to a hyperinflammatory state that can result in severe tissue damage and multiorgan failure. Although hemophagocytosis observed in a bone marrow biopsy can support the diagnosis, it is not always present, especially in the early stages [3].

In rare cases, interstitial lung diseases have been associated with secondary HLH. Sasaki et al. [4] reported such a link, noting the diagnostic challenges due to the overlapping features of systemic inflammation. Our case aligns with this observation, as the chest CT demonstrated bilateral ground-glass opacities typical of AIP, with no other identifiable trigger. Notably, the CT findings did not show features consistent with rheumatoid arthritis-associated interstitial lung disease, such as fibrosis, honeycombing, or airway involvement, further supporting AIP as the likely pulmonary trigger.

This excessive immune response, often described as a cytokine storm, leads to clinical features such as persistent fever, cytopenias, liver dysfunction, and extremely elevated ferritin levels [5]. In our patient, all infectious, autoimmune, and malignant causes were excluded. Her chest CT scan revealed bilateral ground-glass opacities, consistent with AIP [6], which likely acted as the trigger for HLH in this case.

Once HLH was suspected, the medical team acted quickly. They started treatment with pulse-dose steroids and Anakinra, a medication that blocks interleukin-1, one of the key drivers of inflammation in HLH [7].

The positive response to Anakinra aligns with findings by Eloseily et al. [7], who demonstrated its benefit in pediatric HLH, and La Rosée et al. [8], who support its use in adult HLH triggered by autoimmune or inflammatory conditions.

**Figure 3:**
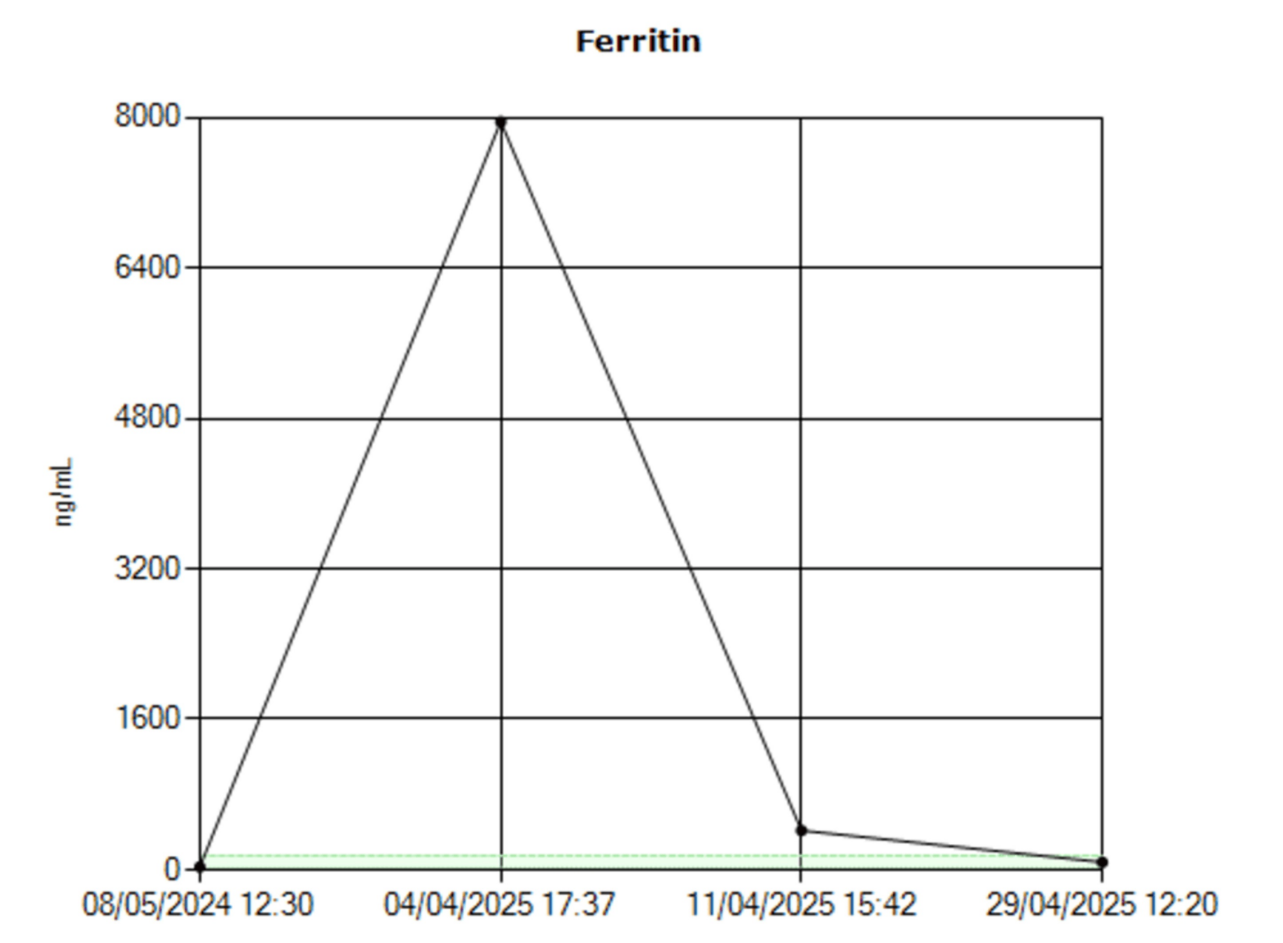
Ferritin trend.

**Figure 4:**
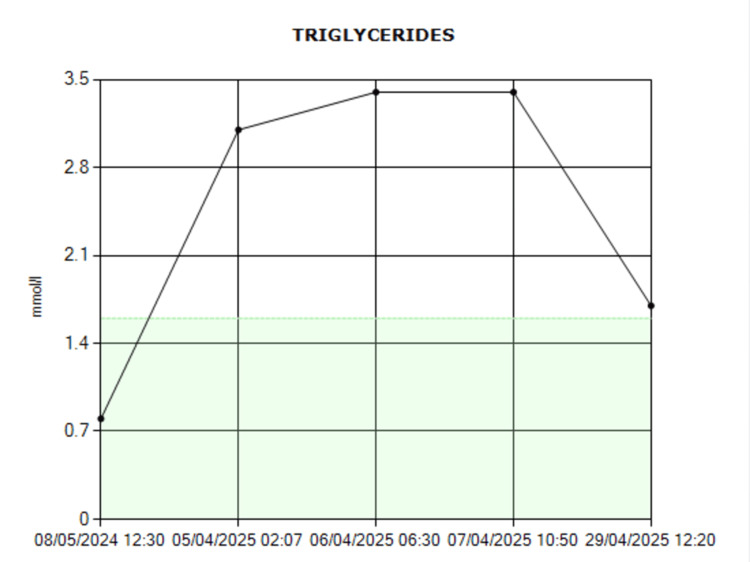
Triglycrides trend.

While some cases of HLH require aggressive chemotherapy, this patient responded well to immunosuppressive therapy alone, likely due to early detection of the condition [8]. The patient demonstrated a marked improvement in laboratory parameters following treatment (Table 2). Ferritin levels showed a rapid decline from 7597 ng/mL to 80.9 ng/mL (Figure 3) and triglyceride levels decreased from 3.4 mmol/L to 1.7 mmol/L (Figure 4).

**Table 2:** Laboratory test results after treatment.

Test	Result	Normal Range
Ferritin	80.9 µg/L	13-150 µg/L
Triglycerides	1.7 mmol/L	0-1.6 mmol/L
Hemoglobin	123 g/L	120-160 g/L
Platelets	304×10⁹/L	150-450×10⁹/L
Fibrinogen	2.8 g/L	1.8-3.6 g/L
White blood cell count	9×10⁹/L	4-11×10⁹/L
ALT (alanine aminotransferase)	50 U/L	0-32 U/L
ALP (alkaline phosphatase)	93 U/L	30-130 U/L
AST (aspartate aminotransferase)	23 U/L	0-32 U/L
C-reactive protein	0.8 mg/L	0-4.9 mg/L
International normalized ratio (INR)	1	0.8-1.2
Albumin	37 g/L	35-50 g/L
Creatinine	50 µmol/L	44-80 µmol/L

Compared to other published cases, ours is unique due to the combination of (1) a rare pulmonary trigger, (2) the absence of hemophagocytosis in the bone marrow, and (3) full recovery with steroids and IL-1 inhibition alone. These distinctions suggest the need for a broader diagnostic lens and reinforce the evolving understanding of HLH presentations, as noted by Jordan et al. [9].

Without treatment, HLH has a high risk of death, reported to be as high as 70% in some studies [1]. Early recognition and intervention can change the outcome dramatically. This case serves as a powerful reminder that HLH should be considered when patients present with systemic inflammation, even if the cause is not immediately obvious [9].

## Conclusions

This case underscores the critical importance of considering HLH in the differential diagnosis of patients presenting with unexplained fever, cytopenias, and markedly elevated inflammatory markers. Although HLH is rare, particularly when associated with AIP, a rapidly progressive and idiopathic interstitial lung disease characterized by sudden respiratory failure, its potentially fatal outcome necessitates a high index of suspicion. In this young woman with underlying rheumatoid arthritis, the early clinical recognition and swift initiation of immunosuppressive therapy were key to her favorable outcome. Her complete recovery without the need for cytotoxic agents illustrates that early intervention with corticosteroids and cytokine inhibitors like Anakinra can be life-saving. This case also highlights the diagnostic complexity of HLH, as patients may not always exhibit all classical features, such as hemophagocytosis in bone marrow. Clinicians should be vigilant in interpreting clinical and laboratory clues in the broader context. Moreover, it is crucial to rule out infectious, malignant, and autoimmune triggers promptly. As awareness of HLH continues to grow, especially in adults, more cases like this may be recognized early and treated effectively. This report serves as a reminder that atypical presentations demand proactive and multidisciplinary management to improve patient survival.
